# A Holistic and Interoperable Approach towards the Implementation of Services for the Digital Transformation of Smart Cities: The Case of Vitoria-Gasteiz (Spain)

**DOI:** 10.3390/s21238061

**Published:** 2021-12-02

**Authors:** Félix Larrinaga, Alain Pérez, Iñigo Aldalur, José L. Hernández, José Luis Izkara, Patxi Sáez de Viteri

**Affiliations:** 1 Mondragon Unibertsitatea, Goiru kalea 2, E-20500 Arrasate-Mondragon, Gipuzkoa, Spain; aperez@mondragon.edu (A.P.); ialdalur@mondragon.edu (I.A.); 2 Fundación CARTIF, Parque Tecnológico Boecillo 205, E-47151 Boecillo, Valladolid, Spain; josher@cartif.es; 3 TECNALIA Basque Research and Technology Alliance (BRTA), Parque Científico y Tecnológico de Bizkaia, Astondo Bidea, Edificio 700, E-48160 Derio, Bizkaia, Spain; joseluis.izkara@tecnalia.com; 4 Corporación MONDRAGON, Pº J. M. Arizmendiarrieta 5, E-20500 Arrasate-Mondragon, Gipuzkoa, Spain; psviteri@mondragoncorporation.com

**Keywords:** urban data platform, IoT and monitoring, digitalisation, added value services, interoperability, smart cities

## Abstract

Cities in the 21st century play a major role in the sustainability and climate impact reduction challenges set by the European agenda. As the population of cities grows and their environmental impact becomes more evident, the European strategy aims to reduce greenhouse gas emissions—the main cause of climate change. Measures to reduce the impact of climate change include reducing energy consumption, improving mobility, harnessing resources and renewable energies, integrating nature-based solutions and efficiently managing infrastructure. The monitoring and control of all this activity is essential for its proper functioning. In this context, Information and Communication Technology (ICT) plays a key role in the digitisation, monitoring, and managing of these different verticals. Urban data platforms support cities on extracting Key Performance Indicators (KPI) in their efforts to make better decisions. Cities must be transformed by applying efficient urban planning measures and taking into account not only technological aspects, but also by applying a holistic vision in building solutions where citizens are at the centre. In addition, standardisation of platforms where applications are integrated as one is necessary. This requires interoperability between different verticals. This article presents the information platform developed for the city of Vitoria-Gasteiz in Spain. The platform is based on the UNE 178104 standard to provide a holistic architecture that integrates information from the different urban planning measures implemented in the city. The platform was constructed in the context of the SmartEnCity project following the urban transformation strategy established by the city. The article presents the value-added solutions implemented in the platform. These solutions have been developed by applying co-creation techniques in which stakeholders have been involved throughout the process. The platform proposes a step forward towards standardization, harmonises the integration of data from multiple vertical, provides interoperability between services, and simplifies scalability and replicability due to its microservice architecture.

## 1. Introduction

Cities are one of the most valuable assets of today’s Europe. They are becoming hubs of modern civilisation [1], with implications for sustainability and more efficient use of resources. Cities have a central role in sustainable development, which is clearly reflected in the Sustainable Development Goals of the United Nations’ 2030 Agenda, which aims to achieve sustainable and resilient cities [2]. One of the main reasons for the great potential of cities in the sustainable development is the population growth in cities, which is observed in the fact that 70% of the population now lives in urban areas [3]. This number is expected to increased up to 85% by 2050 [4]. However, this growth also has implications in climate change. Cities are currently responsible for 60% of greenhouse emissions [3]. The current European strategic plans aim to reduce 55% of these emissions by 2030 and reach climate neutrality (or carbon zero) by 2050 [4]. However, as global figures grow at a steady rate, a diverging pattern is noticed. While Europe’s biggest cities continue to grow, mid-size and smaller cities are reducing their population, often in a strong relation with industrial decay processes. It is clear that these demographic dynamics are strongly linked to the fact that cities also drive the European economy: 67% of Europe’s GDP (Gross Domestic Product) is generated in metropolitan regions [5]. Research has proved that agglomeration effects have limits and the relationship between income and population size becomes negative, suggesting diseconomies of agglomeration due to congestion and other related costs [6].

To achieve the ambitious impact marked by the European strategic plans, cities need to transform themselves into Smart Cities. This can be achieved by applying new concepts in urban strategies, such as electrification of the thermal energy system, sustainable mobility, or penetration of renewable energy [4]. It is also essential to consider the concepts of Smart People and Smart Economy as the core for urban transformation [7]. According to [8], it is necessary to create an environment where six main pillars are considered: Smart Nature, Smart Living, Smart Mobility, Smart Governance, Smart People, and Smart Economy. Within this transformation, the Information and Communication Technologies (ICTs) play a pivotal role [7]. First of all, ICTs act as the support for digitalisation and thus integrating the aforementioned city pillars or verticals. Secondly, by providing tools in the form of urban data platforms that help to implement analytics and extract key performance indicators (KPIs) to make better-informed decisions [7].

To address these challenges, the SmartEnCity project (GA #691883) [9] was born in 2015. SmartEnCity is an innovation European project, whose main vision is to create Smart Zero Carbon Cities by converging both sustainability and inclusive city concepts. To do so, SmartEnCity applies a systemic approach to reduce the energy demand, maximise the renewable energy supply, develop new urban mobility strategies focused on electric vehicles, and involve citizens in this urban transformation [9]. All these actions are supported by an urban platform named CIOP (City Information Open Platform), which is the integrated ICT solution that manage the data and applications used in the project. The SmartEnCity solutions are applied in three lighthouse cities; Vitoria-Gasteiz (Spain), Tartu (Estonia), and Sonderborg (Denmark).

CIOP is at the core for digital transformation of the lighthouse cities. The ICTs are thus considered as cross-cutting enabling technologies used for monitoring and evaluation of the success of measures [9]. Additionally, they are the means for management, control and integration of valuable information, which is made accessible to different stakeholders, and a tool for social interaction. Having this in mind, the primarily objective of the CIOP is the acquisition of data from the energy and mobility verticals. Secondly, it aims to provide persistent storage of data, making it interoperable among verticals. Finally, CIOP exploits data to obtain Added Value Services (AVS) and analytics to support the decision-making processes of the cities [10]. To that end, a standard architecture based on the norm UNE 178104:2017 [11] has been deployed, following interoperability mechanisms [12].

This paper presents the way of implementing and deploying the CIOP in the city of Vitoria-Gasteiz (Spain). The CIOP addresses the challenges of lack of interoperability within the verticals of the city (see Section 3.1), as well as the capabilities for cross-domain analysis, which are indeed common challenges from city to city, as stated in Section 2. The usage of a standard architecture with harmonised data models to couple a set of AVS makes better-informed decision-making processes feasible in the sustainable urban transformation of Vitoria-Gasteiz. The main benefits of the proposed solution lie then in the holistic approach through the integration of the multiple verticals (energy, mobility, environment, citizens, and economy) within an interoperable framework, and under a user-centric approach.

The paper is structured to describe the current background under which cities and digital transformation take place (Section 2). EU initiatives, current practices in other cities, and research results are considered in such analysis. Next, Section 3 aligns the urban transformation strategy of Vitoria-Gasteiz with the integration of the digital tools (i.e., CIOP) and how the designed architecture fits into this strategy. In Section 4, the CIOP features (interoperability and AVS) are explained. These include the co-creation methods used to develop the AVS in the platform. Co-creation methods consider citizens as the core and principal stakeholders of the solutions deployed. Finally, a discussion on the lessons learned and the conclusions are outlined in Section 5.

## 2. Background

This section presents the state of the art on the European frameworks for digital cities. This includes the ICT and standardization strategies for Smart Cities and current practices in the Smart City urban data platforms development. The objective with this background is to identify trends and good practises on platform development across other cities in Europe. As a motivation for the project, this section highlights the benefits of an urban platform, such as the CIOP, in the digitalisation context of cities.

### 2.1. European Framework for Smart Cities

To begin the analysis of the European Commission’s European frameworks and initiatives, mention should be made of the Smart Cities Marketplace [13] which has its origin in earlier initiatives such as the European Innovation Partnership on Smart Cities and Communities (EIP-SCC). Within its working groups, the one dedicated to Urban Data Platforms is pushing for the “adoption of common open urban data platforms so as to reach 300 million European citizens with competent urban data platforms, by 2025 ”. This strategy defines urban data platforms as “solutions that help cities digitise their services and connect across them” [13]. Under this scope, urban data platforms should be designed according to the following features:

- Management of volumes of data coming from multiple and heterogeneous sources;
- Improvement of health and well-being;
- Reduction of the energy consumption by promoting local low-carbon energy;
- Integration of joined-up and multi-purpose services and infrastructures.

Moreover, urban platforms should reach different stakeholders, giving support to cover the needs of end-consumers (i.e., citizens), industry, and public authorities.

**Figure 1:**
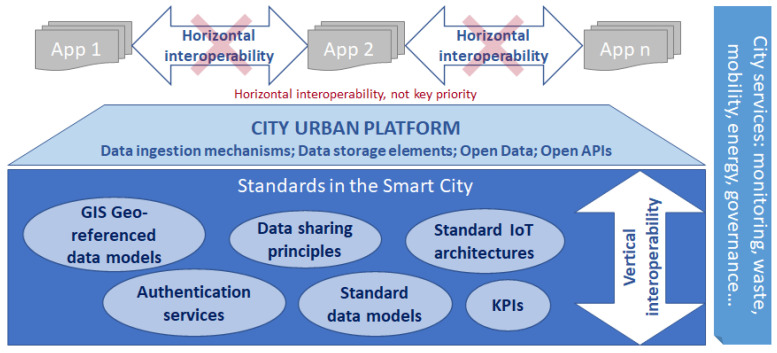
High level overview of the urban platform (approved by the DG CNECT) [14].

In this regard, the EIP has published a technical report that establishes the commons grounds for urban data platforms. These should implement logical architectures to integrate data flows, manage data as open data, whenever possible (i.e., data privacy and protection), and use standards. Bearing this in mind, Figure 1 overviews the high-level reference for an urban platform [14].

According to this high-level overview, the urban platforms should:

- Cater for vertical interoperability (horizontal interoperability is not the priority);
- Enable replicability of the apps, services, and solutions;
- Reduce the management and maintenance costs;
- Enable real-time capabilities;
- Provide open APIs (Application Programming Interfaces) and SDKs (Software Development Toolkit) for data sharing.

#### ICT and Standardization Strategy for Smart Cities

In line with the European Commission (EC) initiative, the EIP-SCC workstream 2 has published a reference architecture in order to standardise the urban platforms development. It is based on a set of capabilities distributed in layers (from 0 to 7 according to the numbering of [15]) and is split into eight layers, as follows [15]:

0. Field equipment capabilities to connect the external environment (IoT, devices...);
1. Communication capabilities to enable the exchange of data;
2. Device asset capabilities to support device integration;
3. Data management capabilities to make use of the gathered data;
4. Integration and orchestration capabilities;
5. Generic city capabilities to enable the deployment of generic city services;
6. Specific city capabilities to enable the deployment of specific city services;
7. Stakeholders capabilities to collaborate and engage citizens.

**Figure 2:**
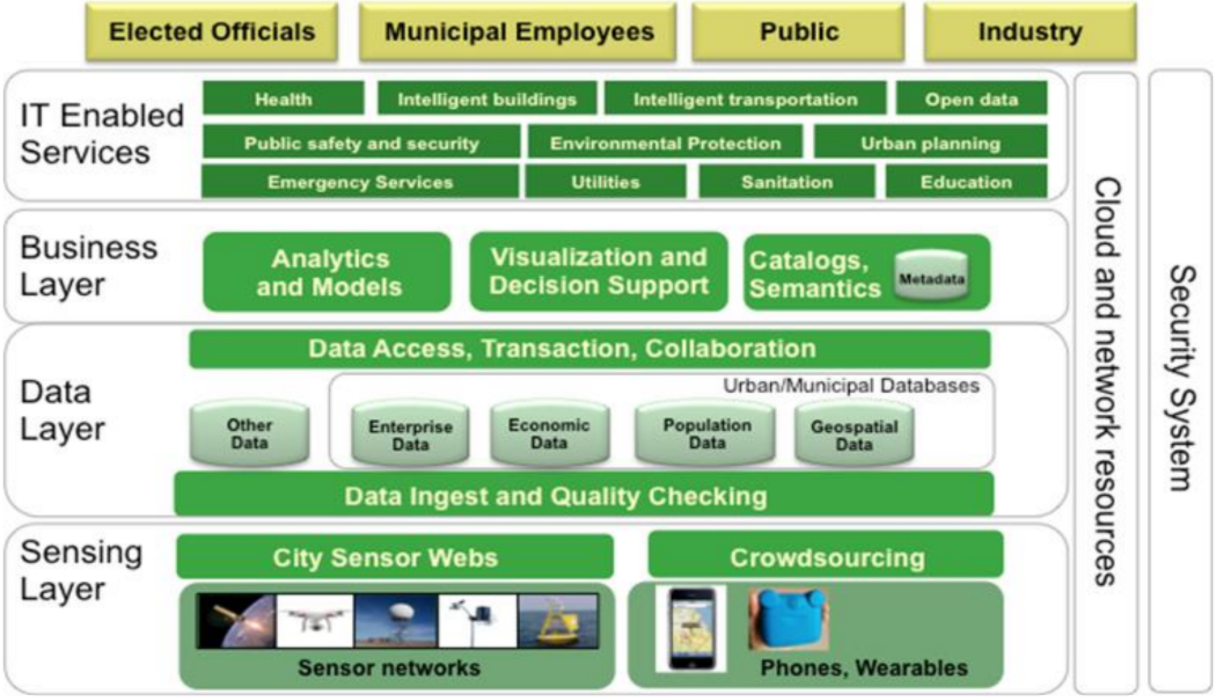
ESPRESSO urban platform architecture reference [16].

It is important to highlight the complexity of the proposed reference architecture, which holds many subcategories for each capability. With the aim of reducing this difficulty, the project ESPRESSO proposes a system approach to standardisation, targeting replicability and reusability [16]. ESPRESSO defines the architecture depicted in Figure 2, which simplifies the EIP approach to four main layers: Sensing, Data, Business, and Services.

In addition to these initiatives launched by the EC, there are other standards to be taken into account. The first one is the norm UNE 178104:2017 [11], published under the umbrella of the ITU-T (International Telecommunication Union of Telecommunications). The norm is aligned with the previous recommendations, but includes an interoperability layer as an important aspect for urban data platforms. Another example of standardisation is the norm DIN SPEC 91357 [17], which focuses primarily on data, providing interfaces, processing, and integration with the objective to share smart data through the services. Finally, ISO 37120 [18] establishes models about how to manage and show data in dashboards.

After this analysis of standards, norms, and recommendations, it could be stated that the management, collection and exploitation of data from apps and services are common characteristics from most reference architectures. A very important aspect to be addressed is interoperability, which is identified as a key factor in UNE 178104 standard. Interoperability is the main reason for the selection of this standard as the reference architecture for the CIOP. In other words, the CIOP has been implemented following the guidelines and recommendations given by the UNE 178104:2017 [11]. Consequently, it can be stated that the CIOP has been developed according to standards and considering interoperability from the design phase.

### 2.2. Current Practices: Other Urban Platforms and Researches

Cities constitute complex sociotechnical systems where the needs of citizens, social entities, and governments are combined [19]. Ideally, a smart city model is based on the integration of different domain-oriented technological developments, under a unique digital context in the form of a platform. Urban Data Platforms are at the core of the digital transformation and the basis for data-driven solutions addressing the challenges of today’s cities and communities [20].

There are several initiatives working in the definition and implementation of urban platforms. For instance, the authors in [21] point at the importance of vertical interoperability by re-defining an open specification framework under which urban platforms are developed. It analyses the previous European initiatives (Section 2.1) to combine the advantages of them. However, as it is a redefinition, it does not follow a standard approach and is therefore not compliant to standards. Moreover, although it fosters an holistic perspective, there is still no consensus about this view. There are several research projects working on specific city verticals. This is the case of [22,23], who propose platforms for waste and noise management, not considering the cross-cutting effects, such as the impact of mobility in noise levels of the city.

Since 2014, the EC has funded between two and four smart city lighthouse projects annually through the SCC1 H2020 call. This amounts to a total of 17 projects until 2019, involving 46 lighthouse cities and 70 partners from all over Europe [24]. One of the main technological challenges addressed in these projects is to ensure a common ICT reference architecture for Smart City implementation projects. However, there are as many different platforms as there are cities and communities in Europe. The proliferation of smart city initiatives across Europe, is creating a number of urban ‘living labs’ where pilot projects can be tested and evaluated. Outside Europe, Asia, and America are the continents with the largest number of Smart City implementations reported [25]. High-income countries, such as the United States and China, have a high number of smart city deployments in different locations. While other continents and cities have fewer smart cities, it is only a matter of time before more emerge, following in the footsteps of their predecessors [26].

Pellicer et al. [27] summarized the various smart city works or projects across world cities under different smart city domains: smart governance, smart mobility, smart environment, and smart living. Some of the most relevant urban platform implementations are highlighted next. The Smart Valencia (Spain) project [28] implements Valencia’s urban platform from a holistic perspective to manage data across multiple domains and extract KPIs to support decision-making processes. Although the platform shares applications with the citizens, co-creation strategies have not been implemented. In this way, OrganiCity [29] covers this gap by establishing a co-creation framework to develop city digital solutions to solve urban challenges (i.e., urban data platform), but the framework lacks standard guidelines to be applied for interoperable cities. SynchroniCity [30] develops a common concept for digital and interoperable cities driven by IoT and data. The project considers large pilots such as Bordeaux (France), Helsinki (Finland), or Santander (Spain) with great potential. However, again, SynchroniCity is led by use cases considering specific verticals, missing the holistic view of the city. Consequently, Santander which is part of this initiative, relies on Smart Santander [31] to implement other pillars such as transport and tourism. The combination of these initiatives still lacks verticals such as energy, which is quite important from a sustainable transition point of view.

An important aspect to consider when developing smart city solutions is to involve citizens in their design, testing, and validation. According to [32], the digital public services must meet the expectations of the new wave of users that can no longer dissociate their daily activities from the large-scale use of smart technologies. On the other hand, [33] identifies that although most authors agree on the importance of citizen’s participation, the co-creation process does not always include non technological stakeholders. The paper also presents a brief review of citizen engagement in the design of smart city solutions. [34] goes even further, considering that tracking of behavioral and psychological users’ input allows studying the impact of solutions on the users’ daily habits and consequently move towards an active engagement of citizens in a hybrid techno-social manner. Co-creation of solutions during the whole life-cycle should be common practice in the development of smart city applications.

### 2.3. Motivation: SmartEnCity CIOP beyond the State of the Art

From the current practices stated in the previous sections, there are some aspects, whose combination is identified as a progress beyond the state of the art and has been the motivation for the project:

- Making use of standard architecture for urban data platforms, which promotes re-usability and replicability;
- Providing a one-stop-shop concept for integrating the verticals of the city so that the city services could be accessed from a holistic and cross-domain perspective;
- Establishing standard and harmonised data models and data sharing mechanisms that ensure interoperability, not only between verticals of the city, but also with external tools making data available for entrepreneurship;
- Provide common architectures and reference models that support standardisation for smart cities;
- Incorporating AVSs, which allow better management of the city and provide additional value to the data obtained;
- Involving both citizens and other relevant stakeholders participants on the design of solutions, keeping them engaged in the digital transformation of the city;
- Assuring scalability, extensibility, and upgradeability of components and frameworks. This is essential for new deployments, enhancements, or seamless migrations of smart city platforms.

Considering these aspects, this paper presents the innovation steps followed towards the digitalisation of the city of Vitoria-Gasteiz within the SmartEnCity project. The CIOP is the ICT solution that supports the implementation of the “Digital Vitoria-Gasteiz”. Before we outline the implementation of the urban platform CIOP, it is necessary to present what is the sustainability strategy for Vitoria-Gasteiz and the initial situation of the city. This is addressed in the following section where the CIOP reference architecture and the citizen engagement strategy are also outlined.

## 3. Sustainability Strategy in Vitoria-Gasteiz: The Role of Digitalisation

As stated before, the SmartEnCity project aims at an adaptable and replicable approach to the urban transition towards sustainable, smart, and resource-efficient cities in Europe [9]. In particular, one of the cities is Vitoria-Gasteiz, which is the capital of the Basque Country (Spain). It has a population of 249,000 inhabitants gathered in an area of 276.81 km^2^. Vitoria-Gasteiz offers 42 m^2^ of green space per person with around 20% of the municipal area urbanised. Vitoria-Gasteiz, as the rest of the Basque Country, relies almost completely (more than 90%) on the import of fossil fuels both for direct use (natural gas, petroleum products), and for electricity generation.

Primary Energy Consumption in the city is not available due to industrial consumption not being recorded, however, the total building energy consumption per year in 2013 was 1823 GWh/year, where the residential sector amounts for 1107 GWh/year, while 84 GWh/year are due public buildings. The distribution of consumption by energy sources in 2006 showed that 44.7% was derived from petroleum, followed by a 26.8% natural gas and 25.9% of electricity. Global Warming Potential (GWP) per capita is 3.1 Tn equivalent CO_2_/year capita without considering the industrial sector.

Because of these reasons, Vitoria-Gasteiz has realized the following interventions within the SmartEnCity project: energy retrofitting of buildings in the Coronación district (25 buildings out of the 15,326 in the metropolitan area), deployment of a biomass-based district heating for these 25 buildings and fostering clean and sustainable mobility through 13 electric buses fleet. The final target is to become a Smart Zero Carbon City (SZCC). SZCC considers a resource-efficient urban environment where the carbon footprint is eliminated by keeping the minimum energy demand; supplying renewable energy; and making citizens aware of climate change [35].

To do so, urban strategic plans become pivotal [36]. These are evaluated by means of quantitative indicators [35]. In this way, Vitoria-Gasteiz has prepared its own sustainable transformation (named Cities4ZERO), consisting of the followings steps [36]:

1. Engage: Involving all the relevant stakeholders;
2. Analyse: Gathering city information and indicators;
3. Diagnose: Evaluate the indicators [35];
4. Envision: Co-create the future vision of the city per domain (e.g., energy, mobility...);
5. Plan: Planning strategic areas;
6. Integrate: Cross-sectoral strategic plan.

The digitalisation of city assets then becomes necessary for the urban transformation, which is realised by means of the CIOP. The way how the CIOP supports the SZCC strategy is based on the next four pillars:

- Monitoring of the performance of the systems, e.g., energy demand of buildings, and gathering data from IoT assets of the city;
- Evaluating through well-defined indicators in order to extract the impacts of the actions and the lessons learned;
- Integrating the multiple pillars (e.g., energy, mobility, etc.) of the city to make them “interoperable” and providing a holistic view;
- Managing assets to increase the performance of the systems.

### 3.1. Digital Vitoria-Gasteiz: Initial Situation

At the beginning of the SmartEnCity project, Vitoria-Gasteiz did not have an urban platform. The city had several monitoring systems for urban elements and services, which were used and maintained by the concessionaire of each urban service. They were the result of isolated initiatives by different departments of the municipality. Among them, it is worth to highlight: A traffic control and management system; a real-time location system for the city bus fleet; a weather and air quality information system for the city’s main urban spaces; and a monitoring system of the energy behaviour of municipal buildings and remote control of their energy systems.

To support these monitoring systems, the city of Vitoria-Gasteiz had deployed an optical fiber network as the base infrastructure for the connectivity of different municipal systems. A WiFi network infrastructure was set up as well, both in urban spaces and public buildings.

In a further step towards the Smart City paradigm, the city of Vitoria-Gasteiz developed a public space maintenance platform named “Via Digital”. This platform made it possible to coordinate different municipal services with competence in urban spaces (e.g., parks and gardens, public lighting, traffic signals or road pavement). It integrates an automatic irrigation remote control system for parks and gardens in the city. The public lighting of the historic city was also monitored and controlled point-to-point by a telemetry system. In the same way, the city council had a platform for the management and control of garbage and street cleaning with containers and collection trucks monitored in real time to adapt the routes.

In terms of citizen involvement and engagement, the Vitoria-Gasteiz city council also owned a website where citizens could contact and carry out e-procedures. It is a single point of access where a citizen, through digital signature, has a mailbox for suggestions and other interaction procedures with the administration. Finally, Vitoria-Gasteiz is committed with transparency by using international standards and open data for the reuse of the information generated.

### 3.2. CIOP Reference Architecture

The implementation of the digital city of Vitoria-Gasteiz is based on a standard reference architecture. Here, standardisation is remarkable as it deals with the SmartEnCity overall objectives of replicability across European cities [9]. For this, UNE 178104:2017, as defined by the Committee for Smart Cities (CTN) 178 [11] was selected. The main advantage of selecting this framework are:

- Standard implementation of digital Smart Cities that fosters replicability;
- Interoperability through the corresponding layer to promote the holistic integration of verticals’;
- Flexibility in the data management, allowing multiple data repositories and data models to merge data-sets and extracting knowledge from data.

**Figure 3:**
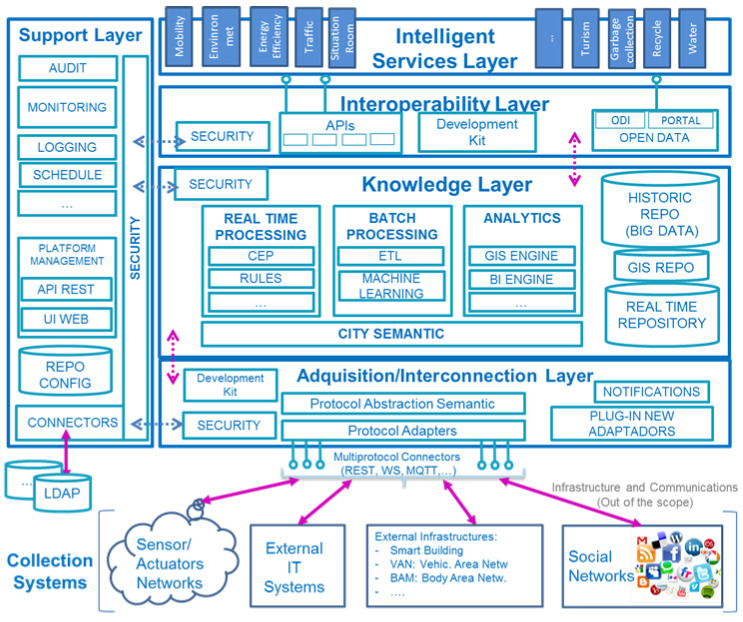
UNE 178104 Reference Architecture [11]. (Source: UNE 178104:2017 Sistemas Integrales de Gestión de la Ciudad Inteligente. Requisitos de interoperabilidad para una Plataforma de Ciudad Inteligente (https://tienda.aenor.com/norma-une-178104-2017-n0059471). Reproduced with the authorisation of AENOR. )

The scheme is presented in Figure 3 [11]. It is distributed into five main layers:

- Collection Systems layer, related to all the infrastructures that provide data, such as IoT sensors (SCADAs and PLCs), external information systems, social networks, etc;
- Acquisition/Interconnection layer, which implements the adapters for the protocols to collect the data from the sensor network and acquire the necessary information;
- Knowledge layer, where data models and management (repositories) are deployed for data analysis using ETL mechanisms (Extraction, Transformation, and Loading);
- Interoperability layer to facilitate the exchange of information between different parties through common representation models. This layer is related to the concept of Open Data;
- Intelligent services layer where AVSs are found, ranging from energy efficiency or mobility services to governance services. It is precisely in this last layer where the digital service is included and, in particular, within the energy efficiency vertical.

### 3.3. Engagement of Citizens: Co-Creation Strategy

The urban strategy relies on a digital transformation, but considering the citizens as an important player (step “engage” of SZCC) [36]. Hence, engagement should also be applied on the development of the CIOP. This section presents the co-creation methodology, while Section 4.1 will describe how it has been applied in the city of Vitoria-Gasteiz.

The main principles of the co-creation methodology are based on the User-Centred Design (UCD) [37]. UCD deals with involving end-users throughout the development process of a solution, including the design stage, to consider their preferences and constraints. It follows the ISO standard for the design of human-centred interactive systems [38]. The methodology has been adapted to the Smart Cities context by applying the User Driven Innovation methodology proposed by the Design Innovation Centre [39]. In addition, the methodology incorporates guidelines from [40] to identify stakeholders and determine which questions are key to understanding the type of data stakeholders need and the solutions they will use.

The methodology proposes a process composed by four phases:

- Phase 1—Conceptualization: It contextualises the problem to be solved, e.g., sizing the context and the objectives of the application, identifying the actors, proposing solutions, visualising technologies and channels to develop these solutions, etc. It includes a set of steps:
  - Identification of users and their profiles (e.g., consumption habits);
  - Brand value identification to establish the project’s identity;
  - Identification of the technological framework and the tools to be used;
  - Value proposition.
- Phase 2—Design: It identifies how and when the user will interact with the system, decision-making strategies, user-friendliness features, and data sources and information flow. The activities of this phase are:
  - Experience design: User stories describing the context and the steps the user will take when using the system;
  - Static design: Graphical interface, solution’s information architecture, etc.;
  - Analytical design: Identify the sources of data and the treatment of information by all the actors involved in the system;
  - User testing: Contrast the design with potential users of the system, identifying the weak points of the solutions before being implemented;
  - Technical requirements: Identify the technological solution in detail in order support developers in the implementation.
- Phase 3—Development: Implementation of the system, in terms of infrastructure and software. It translates the previous requirements to software assets. It includes the following items:
  - System and architecture;
  - Software development;
  - Integration and testing.
- Phase 4—Deployment: Make the solution available to stakeholders in a production environment. This stage also addresses the analysis and monitoring of the system created, with the aim of checking its operation and collecting information to determine the validity of the results. This phase covers the following tasks:
  - Deployment;
  - Monitoring of performance metrics.

## 4. CIOP Implementation and Deployment

Based on the reference architecture and design guidelines of the previous section, this section describes how they are implemented to build the digitisation platform of Vitoria-Gasteiz, the CIOP platform. One of the main objectives of the CIOP platform is to provide AVS applications that support stakeholders in achieving their sustainability, energy efficiency, and mobility goals. CIOP relies on different data structures to manage information. The most relevant data structure available in CIOP is the Hadoop framework for Big-Data management. The Hadoop framework is a distribution of Hortonworks that is deployed on a cluster of 4 servers with a total memory of 40 GB and a storage capacity of 4 TB. This framework supports the implementation of AVSs enabling the analysis and management of data in order to deliver high value services that enable the stakeholders (identified in Section 4.1) to achieve their objectives. First the section outlines the implementation of AVS using the UCD methodology presented in Section 3.3. This shows the life cycle of an application in its development. The following subsections present the journey the information makes through the platform considering the layers of the reference architecture. That is, it explains how the information is collected, stored, aggregated, and used by the AVS. The subsection addresses data ingestion mechanisms, data models, and repositories for interoperable and holistic data storage, and finally, APIs for sharing this data.

### 4.1. User-Centred Design

The involvement of the key players in the development and usage of the CIOP is crucial, while assuring engagement in the digital transformation [36]. The methodology described in Section 3.3 has been applied to realise the AVS deployed in the platform. For each AVS, the methodology responds to the next questions:

- Who are the most relevant stakeholders?
- What information should be communicated to stakeholders?
- How should this information be presented?
- What is the best communication channel and where should it be located?
- What makes stakeholders interact with the solution?

These questions refer not only to technological aspects but also to communication and user behaviour issues. The application of the methodology in each step and the results obtained are summarised next. During the Conceptualization phase we mainly identify the stakeholders for each AVS, find the ideal solution and propose the technological framework to build it. Thus, the following stakeholders were identified:

- Residents as agents interested in their energy consumption and comfort conditions, which need to be informed about recommendations and good habits (awareness). To be successful with these stakeholders it is essential to create solutions that encourage them to participate and take part in cost-saving measures and sustainability activities (engagement);
- Energy Service COmpany (ESCO) as stakeholders interested in offering energy and comfort services to their clients (residents, communities, cities, etc.). These stakeholders demand information such energy demand, production forecast, energy ratings, building capacity and comfort conditions among other KPIs;
- Mobility managers, who are stakeholders working for transport companies that need dashboards to monitor the performance of their transportation fleets by measuring consumption, CO_2_ emissions, status of charging stations, location of vehicles, etc;
- City managers are agents that alike previous stakeholders, need dashboards to make decisions in relation to energy consumption of public buildings, status and performance of public transport or impact of sustainability measures in the environment.

In order to propose a solution during this phase, working sessions with the different stakeholders were organised to analyse different scenarios, identify alternatives and prioritise with the stakeholders the highest impact solutions.

In the next phase (Design), user stories for the solutions obtained during the Conceptualization phase were created. Mockups were built and validated, along with the stakeholders, by means of tests. Each user story was analysed to identify the information flow from the source to the final service, as well as its usage in the application. The main result for this phase is the technological brief of each AVS that includes the requirements for the solution, the technological elements to be used, the information architecture, the interfaces to communicate with the stakeholders, the data sources, and the content required to implement the applications.

The Development and Deployment phases are specific of the solution to be provided and the final results in the form of AVS are presented in the following subsections. Each development team collects the technological brief and constructs the solution according to the specifications. For the AVS presented in this paper, different environments were considered, and continuous deployment techniques were implemented. There are two existing environments; development and production. The first one is used to implement new functionality and changes that need to be validated before deploying them to the production environment where the final solution is available to stakeholders. Pipelines were used for continuous deployment. They included testing and validation of the new functionality before automatic deployment to the production environment. Gitlab Runner and Docker technologies support the continuous deployment and integration.

Then, identified stakeholders within this co-creation strategy are the main actors for the provision of data (see Section 4.2) and final users of the AVS (see Section 4.5). For instance, resident groups benefit from the social awareness and engagement services (Section 4.5.5) in charge of allowing the monitoring of dwellings, while city managers open data from city assets to be able to continuously analyse the city status based on KPIs (Section 4.5.1). ESCOs use information available in Section 4.5.2 and Section 4.5.3 to make informed decisions about energy and comfort conditions in the buildings/dwellings.

### 4.2. Data Ingestion Mechanisms

**Figure 4:**
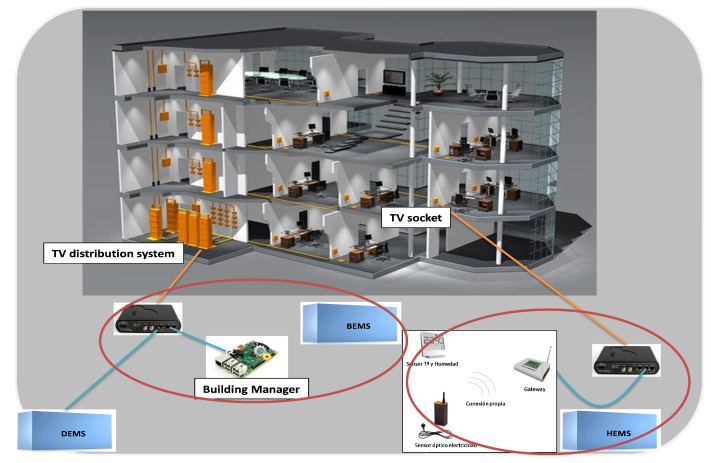
Sensor and device infrastructure at dwelling level.

The CIOP presents different ways to collect and ingest data, including multiple variety, velocity and volume (3 out of the 5 V’s of Big-Data). The mechanisms used here belong to the Collection Systems and Acquisition/Interconnection layers of the UNE 178104:2017 standard [11]. The data sources that are considered in the CIOP are the following ones:

- Thermal energy consumption of buildings;
  - Individual gas boilers measurements taken before the deployment of the district heating. Those data were acquired from Excel sheets provided by the gas network distributor (Nortegas). Mechanisms based on climate conditions were applied to reduce granularity up to daily energy consumption at building level (25 buildings) during 5 years, having 250 KB/year;
  - District heating data obtained by interfacing the Supervisory Control And Data Acquisition (SCADA) and Programmable Logic Controllers (PLC) in BACnet protocol with periodicity of 15 min. At the moment of writing this paper, the integration of the district heating in the 25 buildings just finished, without having historic data yet;
  - Energy demand as simulated energy for all non industrial buildings in the city, being theoretical energy that is needed for each building to comply with comfort parameters. It is obtained from a simulation tool software that generates data in csv format, including average results of heating and cooling demand and solar potential.
- Comfort (temperature, relative humidity and CO_2_) and electricity consumption parameters. 250 dwellings from 25 buildings in the lighthouse district of Coronación are monitored by means of sensors. In each dwelling we have installed the following sensors: 1 energy sensor, 1 temperature sensor and 1 humidity sensor. In 35 dwellings a CO_2_ sensor has been added. This sensor includes additionally a temperature sensor and a humidity sensor. Measures for each sensor are collected every 5 s. Hourly aggregates of all sensor measurements are made and the ASHRAE comfort values are calculated for each dwelling. See Section 4.5.3 for comfort calculation details. Sensors are deployed, taking advantage of the building’s infrastructure. This original architecture for data gathering is based on the TV distributed coaxial system. To achieve that, adapters are placed at the TV distribution system header and each of the dwellings. Figure 4 shows the infrastructure. Thus, the TV distributed system is used to direct that information to the CIOP platform without requiring an Internet connection from each of the dwellings residents. Internet connectivity is provided at building level to route data from each of the dwellings towards the CIOP platform. The network created by this infrastructure is bidirectional and enables the provision of other services to/from the dwellings.
- Electromobility.
  - Public eBuses. A REST-API (JSON format) is provided to collect data from the Web application where data are registered. Here, travelled distance, speed, used energy, regeneration and auxiliary systems’ parameters, among others, are collected from 13 eBuses of the public fleet. Different granularity is offered: activity (each time the eBus starts a trip), daily summary or total aggregation in the full operation period, accounting for 130 KB/day of data;
  - eCar fleet from the Municipality and the district heating operator in Excel format. From the municipal fleet, 2 eCars data are collected in a monthly basis, having only available energy usage, distance, bookings, and users, accounting for 28 KB/year. In the case of the district heating operator, a daily summary of the parameters: distance, energy usage and performance of the energy system is gathered, accounting for 113 KB/year;
  - Rental eBikes, which only gathers monthly summaries of the uses and distances in Excel format;
  - Charging stations associated to each of the EVs listed above. The format and granularity of the data follows the same structure as its associated EV.
- Geographical data represent the 2D and 3D geometry of the different city elements as well as their semantics. For the generation of the 3D urban model based on CityGML the following data sources were used:
  - Cadastral data: GIS file including dimensions and geo-referenced attributes of the city buildings and urban parcels (15,326 buildings);
  - Digital Surface Model (DSM): LiDAR file with elevation data of urban elements. This model contains elevation information of buildings and other constructions (1 m${}^{2}$ resolution);
  - Digital Terrain Model (DTM): LiDAR file with elevation data of the ground, representing the base level of urban elements (1 m${}^{2}$ resolution).
  Buildings are generated in CityGML LoD2 (15,326 buildings), where DSM and DTM were used for estimating real heights of the buildings. Energy simulation results provide the semantic information of the 3D model.
- Social aspects. Citizens should be continuously engaged with the different measures applied in the city. To gather data from them, questionnaires and surveys are used both in Web forms and hand-written.

As described, data come from multiple sources in heterogeneous formats. In order to ingest these data-sets, the CIOP provisions an acquisition and interoperability layer based on the REST API architecture. RESTful web services are offered to ease access to data repositories from the data collection methods, using JSON documents. Each data-set is therefore translated into a CIOP-compliant JSON document, from which data is extracted and stored in the CIOP repositories (see Section 4.3). In addition, the platform is prepared to collect information in the form of messages. A message broker is available, enabling an event-driven architecture based on publish/subscribe or similar message management.

### 4.3. Data Models for Interoperable and Holistic Data

Ingested data is persisted in the CIOP to make it available to services through data sharing APIs, promoting in that manner open data principles. The data models follow the recommendations and structures proposed in the Knowledge layer of the UNE 178104:2017 standard described in Section 3.2. The data on the CIOP platform are structured in several repositories that host data-sets according to their nature. Thus, there are repositories for:

- Time Series Data. This repository stores timeseries measurements coming directly from comfort sensors (5 s frequency). The repository uses InfluxDB, which is a open-source database oriented to IoT deployments. As of today, the repository hosts 2.3 GB of data;
- Structured/relational Data. Relational databases, such as MySQL, PostgreSQL, or MariaDB, are used to manage aggregated information related to dwellings, buildings, districts, mobility, social, and KPIs. Whereas data velocity and volume for dwellings, buildings, districts, and mobility were explained before, the case of KPIs saves daily calculations. At the moment of writing this paper, the repository stores 670 MB of structured/relational data. Among those data, 31 KPIs are being calculated in the pillars of energy, comfort and mobility. ICTs, Life Cycle Analysis and social aspects are not considered since the indicators for these verticals are static, calculated once, and updated many times afterwards;
- Historical data. Complementing the structured data, a historical repository is deployed to keep the persistence of all data-sets. This historical repository contains the structured data from cross-domain databases so as to store permanently in a common space. Two main advantages are addressed: historical data can be recovered in case necessary (data analysis, disaster recovery, etc.) and to release space in the main repositories. To store the historical data, Hadoop Distributed File System (HDFS) is selected, being a scalable, flexible and fast technology;
- Geographical Data. Geographical data about consumption points or vehicles distributed throughout the city are stored in geographic databases included in solutions such as ArcGIS Enterprise and other Environmental Systems Research Institute (ESRI) products;
- 3D Information. Virtual 3D city models are generated using Open Geospatial Consortium (OGC) standard CityGML [41] and stored in XML format. CityGML is an open data model and XML-based format for the storage and exchange of virtual 3D city models, which is interoperable with INSPIRE Building data theme [42]. As the model is very large, for visualization purposes the model is exported to 3DTiles. 3DTiles can be loaded by parts, allowing for faster model load, and not blocking the viewer.

The data management technologies select for the platform are open source solutions. Aggregated data are calculated from the InfluxDB repository using programming tools such as Node-RED. The information obtained in these secondary repositories is offered by means of a REST API to be consumed by the front-end solutions developed or any application that needs that information (see Section 4.5). The integrated data from the different verticals offered through APIs for consumption constitute the Interoperability layer. This layer agrees with the specifications of the UNE 178104 reference architecture and provides a holistic view for the data.

### 4.4. Data Sharing APIs

Data stored in the CIOP platform is shared using APIs, which are language agnostic and based on JSON documents. The mechanisms used are compliant with the Interoperability layer of the UNE 178104:2017 standard (see Section 3.2). These APIs facilitate the exchange of information between repositories and applications and also enable interoperability among different systems and parties. The structure of the services in the CIOP platform is in line with the microservice architecture proposed in [43]. This architecture structures an application as a set of loosely coupled, collaborating services. According to this approach each service is:

- Loosely coupled with other services enabling a team to work independently for the majority of time on their service(s) without being impacted by changes to other services and without affecting other services;
- Highly maintainable and testable, enabling rapid and frequent development and deployment;
- Independently deployable, which enables a team to deploy their service without having to coordinate with other teams;
- Capable of being developed by a small team which is essential for high productivity by avoiding the high communication issues existing in large teams.

As for the data ingestion mechanisms (Section 4.2), the RESTful architecture is applied to build the interfaces provided by those microservices. CIOP web services are based on the RESTful architecture. Web services are the interoperability mechanisms use to share data between services.

To certain extent, this layer is related to the concept of Open Data.

### 4.5. Added Value Services

This section describes the last layer of the CIOP implementation about intelligent services. Here, several verticals are included: energy, mobility, citizens (via comfort and social awareness applications), and decision-making processes based on KPIs.

#### 4.5.1. Decision-Making Support Based on KPIs

The first of the AVSs is the KPI dashboards for decision-support. This is the clear example of holistic perspective of the city, where several verticals take place: energy, comfort, mobility, ICTs (digitalisation of the city), social (citizens), environmental, and economy. At the same time, it provides a tool to quantitatively assess the urban transformation strategy [36]. In particular, it supports the steps “analyse”, “diagnose” from [35], and the final evaluation through well-established and measurable indicators. Thus, multiple city perspectives can be analysed from a holistic point of view and evaluate different aspects of the city from a single access point.

**Figure 5:**
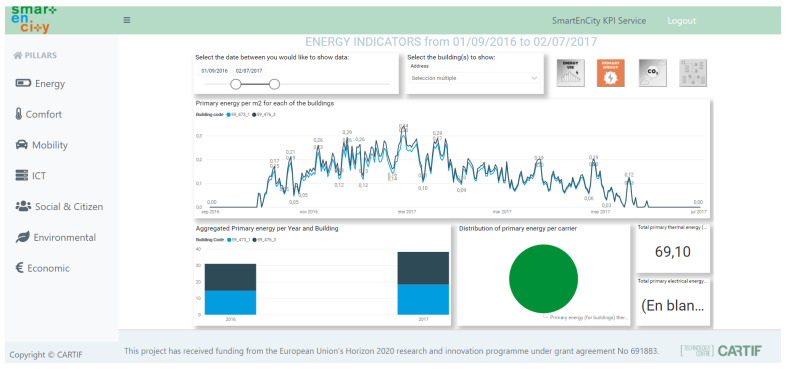
Dashboard for the energy indicators for selected buildings in the period of September 2016–June 2017.

The service gathers raw data from the multiple data repositories of the city, which were defined in Section 4.3, calculates the KPIs, and stores them into the KPI database. Then, from these calculated indicators, a dashboard is created to visualise the results in an user-friendly way. The design of the dashboards has also followed the co-design principles described in Section 4.1 in order to establish the common view with the decision-makers and urban planners. The result is depicted in Figure 5 for primary energy related to buildings. Here, the urban planer or decision maker may observe the trend for the energy demand of the buildings, the distribution of the mix of energy (in the example, 100% of the energy comes from gas) and the comparison between these buildings at an annual level. This example dashboard allows identifying buildings with energy efficiency levels performing below standards to plan renovation roadmaps for energy-efficient buildings and/or districts. Similar strategies would apply to mobility, digitalisation, or social aspects.

#### 4.5.2. Energy-Related Services

Energy is one of the main pillars in the urban transformation deployed in Vitoria-Gasteiz during SmartEnCity project. The main actuation in this way was the retrofitting of buildings to increment the energy efficiency. Under this scope, the GIS3D Viewer for KPI visualization is an added-value service that facilitates the representation and visualization of some indicators on a 3D geospatial model. This service focuses on the information available at the building scale and urban elements. On one hand, it shows the energy demand (obtained by simulation tools) and, on the other hand, the energy use of the building, aiming at comparing the theoretical and real situations. In addition, the information combines the geospatial distribution of the information of the buildings, including their characteristics, energy behavior based on simulation and solar potential, with the temporal distribution through graphs (e.g., monthly and annual) of the indicators of the monitored buildings.

**Figure 6:**
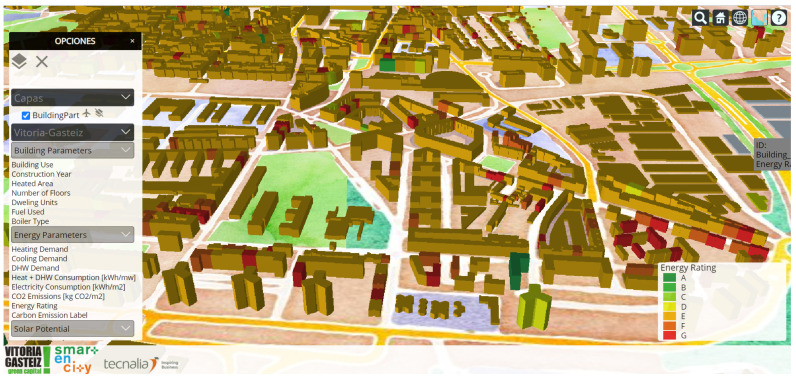
Dashboard for the GIS-based energy services for energy demand and usage visualisation.

To represent all this information, a model of the city in CityGML format is generated from the GIS (Geographical Information System) layer of the city and satellite data. Upon this layer, the energy demand of the buildings is depicted, as well as the solar potential. An example is represented in Figure 6 with the energy rating (i.e., Energy Performance Certificate).

As stated previously, the solar potential for photovoltaics installation is also provided. In this sense, both the LiDAR layer and the cadastre have been used. Considering the total roof area of each building and the incident radiation on roofs with a resolution of 1 square meter, a new visualisation layer is printed with the solar potential, whenever the radiation is greater than a well-established threshold.

Energy is not only related to buildings, but also to city infrastructures. In the city of Vitoria-Gasteiz, a biomass-fired district heating has been placed to heat the buildings. The energy production of these systems is very complex and sometimes over- or under-generation happens. Then, within the CIOP, an additional service is included so as to better plan the energy resources according to a predicted energy demand [44]. Based on real energy use and the KPIs, predictions of energy demand are calculated with the goal of supporting better-informed planning of energy resources. Complemented with the next comfort service, it allows the ESCO to analyse the real needs of the buildings both considering demand and comfort conditions.

#### 4.5.3. Comfort-Related Services

Complementary to the energy demand prediction, the analysis of comfort conditions helps the ESCO to determine whether the constraints for liveable spaces are achieved. Through a dashboard, comfort information at building and dwelling levels are presented. By combining this with the energy services, the ESCO would be able to know the impact of energy saving and retrofit measures or identify anomalous situations.

Building comfort calculations are based on the ASHRAE 55 standard [45], which determines the comfort level based on housing conditions (temperature and relative humidity) as well as contextual parameters (occupancy, outside weather conditions, isolation level, etc,). Two values are calculated: Predicted Mean Vote (PMV), which calculates the comfort level from the sensor data and the Predicted Percentage of Dissatisfied (PPD), which estimates the percentage of people in discomfort under those conditions. For the first value, the closest to 0 the line is, the better the comfort level in the dwelling. For the second value, the lower the value the better.

**Figure 7:**
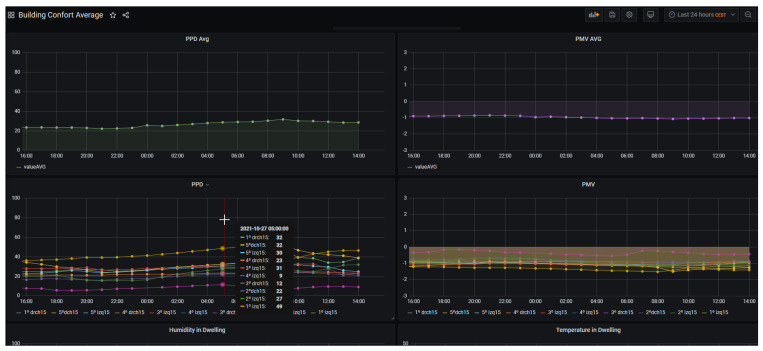
Comfort Dashboard in CIOP.

Based on the data ingestion from Section 4.2, a back-end application built in Node-RED has been developed, together with a Python API, to calculate PMV and PPD according to ASHRAE 55. The front-end or visual interface accommodated in the Intelligence Service layer is developed with Grafana. The AVS dashboard presents several panels (see Figure 7). At the top, building average ASHRAE 55 PPD and PMV values are presented to depict the general comfort conditions of the building. The details for each dwelling inside the specific building are presented in the second row of panels. This enables to compare dwellings among them. Below that dashboard, specific temperature and humidity conditions from a dwelling can be requested in another graph. This allows to determine anomalies of malfunctioning when PMV or PPD values are not available.

#### 4.5.4. Mobility-Related Services

In terms of mobility services, two main AVS are deployed. The first one is a tracking system for the bus fleet. It basically monitors the location (GPS coordinates) of each of the electrical buses in the city of Vitoria-Gasteiz. It also includes instantaneous performance parameters, such as Status of Charge (SoC), speed, or travelled distance.

**Figure 8:**
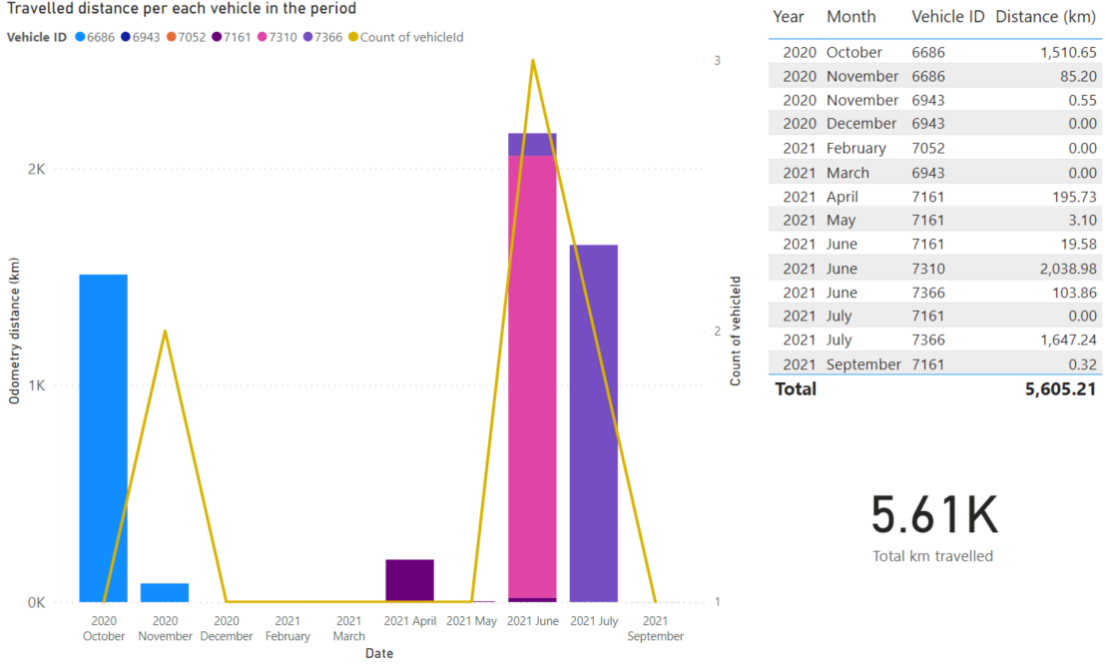
Mobility dashboard for the evaluation of the travelled distance.

The second one is perhaps more interesting from the paper perspective, given that it merges data from different domains, sources, and disciplines. It aims at rendering data analytics for the electomobility assets included in the project so as to extract the environmental benefits (i.e., greenhouse emissions avoided) thanks to the replacement of mobility shifts (e.g., from diesel vehicles to public eBuses or eBikes), as well as energy assessment by additional energy needs due to charging stations. Figure 8 illustrates the example of the dashboard being used during test phase (current stage of the deployment of the eBuses in the city). Here, total travelled distances by buses are observed, in order to obtain in further developments the kg CO_2_ avoided and average duration of the buses activities can be observed together with the energy disaggregation of each vehicle (e.g., energy used by the traction system or energy that has been regenerated) and the supplied energy by the charging stations.

#### 4.5.5. Social Awareness and Engagement Services

Last but not least, as described, the engagement of citizens is crucial. Therefore, tools oriented to the end-consumers or residents need to be available. In this sense, this AVS enables residents to monitor consumption and comfort conditions in their own dwellings in order to keep them informed. The application monitors energy consumption and comfort conditions from sensors installed at home. The main objective is to empower residents, based on knowledge extracted from data, to make their own decisions on managing the energy resources in a more effective way using comfort variables. The application/solution includes:

- Electricity consumption and comfort data collection at dwelling level (Section 4.2);
- Screens with data results and recommendations to the residents (visualization tool);
- Comparisons (before and after interventions) to learn about their performance;
- Usage of relevant indicators for the validation of interventions (KPIs, Section 4.5.1).

Thanks to the infrastructure described in Section 4.2, as well as repositories from Section 4.3, the service makes use of the InfluxDB database to aggregate comfort and energy data from the dwellings. These data are then offered by means of a REST API to be consumed by the front-end or any application that needs that information. The front-end or visualization application provision, in this case, has been developed with Vue.js, which is a JavaScript framework to build Responsive Web Design applications.

**Figure 9:**
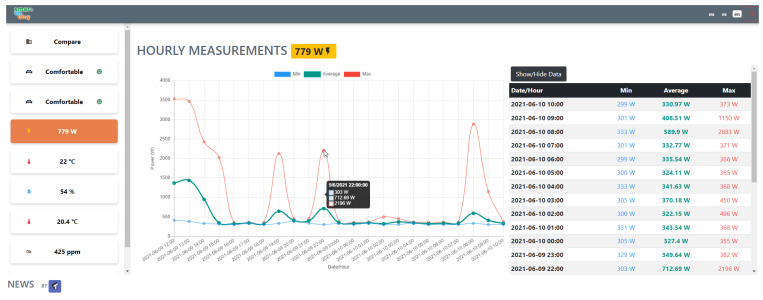
Dwelling Conditions Monitoring AVS.

The service is depicted in Figure 9. Once connected, the residents are presented with a comparison of their dwelling data with the average for both the building and the district. There is an option to select the values for comparison (power, temperature, relative humidity, CO_2_, and comfort), as well as options to view detailed historical information. It is presented both in graphs and tables to allow residents to download. Finally, a set of traffic-light signals complement the visualisation to easily understand current conditions, i.e., green for comfortable, yellow for borderline, and red for under-performance conditions.

## 5. Discussion and Conclusions

This section presents the impact and the main lessons learned in the implementation of the digital strategy in Vitoria-Gasteiz and the development of the urban platform CIOP. For any city, the roadmap to become a Smart City is a long journey, which starts inevitably with the political will to get involved in a deep transformation process for the benefit of its citizens. Getting on board as many municipal areas as possible is key to succeed, to gather both the climate change and commitments from the people necessarily involved, and set a common understanding and knowledge of the obstacles and difficulties that lay ahead. The implementation and deployment of a city platform, a CIOP as described in this work, is a crucial part of that global transformation of the city management. It is not just an ICT Department responsibility but a joint effort from Governance, Civil Works, Contracting and Public Procurement, Citizen Relations, and many other areas. In fact, all municipal areas should collaborate, making its citizens participants during the whole process.

As in any transformation process, the Leader will face reluctance to change from departments and bodies defending the status-quo, and a gigantic effort will be necessary to overcome the inertia of things-being-done-the same-way for years. Fortunately, for our research team, the city of Vitoria-Gasteiz started this journey long ago, with a series of milestones like European Green Capital in 2012, Biosphere Responsible Tourism in 2016 and Global Green City Award in 2019, all in line with its Smart Green City Strategy (or Smart Zero Carbon City (SZCC) as described before).

The CIOP is the path to digitalise the city of Vitoria-Gasteiz. Based on standard architectures (UNE 178104:2017), one of the major benefits is the holistic design, considering the cross-cutting activities within the municipality. That is to say, creating a common environment for multiple verticals of the city: energy, mobility, citizen relations, environmental and ICTs, among other, to comply with the requirements of global transformation. This holistic approach allows better-informed decision-making processes by being able to determine the indirect effects from one domain to another in the city.

Thanks to the standardisation and the creation of common data models across the city, interoperability, as one of the main key findings of this work, is ensured. It is crucial in this global perspective, as heterogeneous data come from various verticals in the city. This is one of the main and current challenges within the digital transformation in the municipalities, where each municipal department is responsible for the management of its data. The solution proposed with the CIOP provides heterogeneous data ingestion mechanisms that accommodate raw data into standard and cross-domain data models.

Once data is made available, which is achieved though the implementation of open APIs, value can be extracted from these data-sets. In the CIOP, this value is extracted in form of added value services (AVS), which are designed to enable better management of the city. The AVS obtain knowledge based on intelligent algorithms and KPI calculation methods, whose results are offered to the identified stakeholders in form of dashboards. As a second key finding, these AVS allow any user to determine the direct and indirect effects of the urban transformation strategies in the city, under a transversal vision. For the city, the CIOP stands for the opportunity to have a central intelligence and data storage system to help build new services, generate additional cross-sectoral decision support systems, real time data availability for better city management, and the opportunity to bring the citizens updated information and communication channels in order to improve their quality of life.

However, citizens should not just be the final users of some applications or services, but the core of the urban and digital transformation. As the third key finding, the consideration of the users from design, under co-creation and co-design methodologies, increments the acceptance and usability of the digital solutions. For instance, in Vitoria-Gasteiz, all the involved citizens accepted the installation of monitoring equipment. Now, residents are informed about their consumption and comfort conditions (empowerment) and can make decisions based on the recommendations received. Other stakeholders of the city also benefit from this approach. For example, ESCOs usually manage energy information coming from their systems and do not know the comfort conditions inside the dwellings. This information is essential and enables them to contrast comfort conditions with the required energy demand of the buildings. Knowledge of the relationship between comfort and energy conditions in households was considered in the early stages of the design of the envisaged solutions. Urban planners are also key partners in this digital transformation and have been involved during the whole development process for those applications related to public buildings, mobility and KPI.

According to all these benefits, one important result to be remarked is the scalability and replicability. An urban platform implementation requires a high degree of complexity that can be inferred from both the backbone services and common framework. This complexity could be a burden when the time comes to service, upgrade, or migrate the CIOP components to a new location or IT service company. The CIOP is designed taking characteristics such as scalability, replicability, and upgradeability into account. Consequently, they are inherent attributes to the system with the objective of easing as much as possible the evolution of the smart platform and the inclusion of additional city services. In this sense, the use of standard architecture, common and cross-domain data models, as well as a microservice structure for the APIs supports the scalability, replicability, and upgradeability of the solution.

The development of the CIOP has also served to validate technologies and infrastructures used in other domains whose application in the Smart Cities domain opens great opportunities for the provision of new services. Among these innovation actions highlights the usage of the TV coaxial infrastructure to create a network that enables bidirectional communication to/from the dwellings and consequently the provision of services at building level. Technologies for presenting information (geographic, energy, KPIs, etc.) in a 3D format enrich the visualisation tools and offer interfaces to citizens that facilitate their interpretation and use. Energy demand forecast of buildings is also possible using weather forecast information, previous measurements and machine learning algorithms such as clustering or data regression.

Currently, all components of the CIOP for Vitoria-Gasteiz are running at the facilities provided for the project. That is, the project is hosting the platform, providing the data storage and running the dedicated services. As primary future work, the smart city platform will be seamlessly migrated to other facilities or hosting services upon the municipal infrastructure. Furthermore, also as future work, the current CIOP approach is limited to a set of city verticals that need to be extended to other domains, such as governance or waste management. Data from these other domains need to be accommodated to the existing data repositories, as well as new KPIs which should be defined and calculated to extend and upgrade the current status of the CIOP. Finally, thanks to the capabilities of extensibility and upgradeability via open data and APIs, new services in the current existing domains are another research line. For example, the exploitation of mobility data for route tracking, car-sharing, or effects in the pedestrianisation of streets.

## Data Availability

Not applicable.

## Abbreviations

The following abbreviations are used in this manuscript:

**API**	**A**pplication **P**rogramming **I**nterfaces
**AVS**	**A**dded **V**alue **S**ervices
**CIOP**	**C**ity **I**nformation **O**pen **P**latform
**CTN**	**C**ommittee for **S**mart **C**ities
**DSM**	**D**igital for **S**urface **M**odel
**DTM**	**D**igital for **T**errain **M**odel
**EC**	**E**uropean **C**ommission
**EIP**	**E**uropean **I**nnovation **P**artnership
**EIP-SCC**	**E**uropean **I**nnovation **P**artnership on **S**mart **C**ities and **C**ommunities
**ESCO**	**E**nergy **S**ervice **CO**mpany
**ETL**	**E**xtraction **T**ransformation and **L**oading
**GDP**	**G**ross **D**omestic **P**roduct
**GIS**	**G**eographical **I**nformation **S**ystem
**HDFS**	**H**addop **D**istributed **F**ile **S**ystem
**ICT**	**I**nformation and **C**ommunication **T**echnology
**ITU-T**	**I**nternational **T**elecommunication **U**nion of **T**elecommunications
**KPI**	**K**ey **P**erformance **I**ndicators
**OGC**	**O**pen **G**eospatial **C**onsortium
**PLC**	**P**rogrammable **L**ogic **C**ontrollers
**PMV**	**P**redicted **M**ean **V**ote
**PPD**	**P**redicted **P**ercentage of **D**issatisfied
**SCADA**	**S**upervisory **C**ontrol **A**nd **D**ata **A**cquisition
**SDK**	**S**oftware **D**evelopment **K**its
**SZCC**	**S**mart **Z**ero **C**arbon **C**ity
**SoC**	**S**tatus **o**f **C**harge
**UCD**	**U**ser **C**entered **D**esign
